# Societal Views on Aging: Comparing Neighborhood Lunch and Lifelong Learning Program Participants

**DOI:** 10.1093/geroni/igaf122.303

**Published:** 2025-12-31

**Authors:** Su-I Hou

**Affiliations:** University of Central Florida, Orlando, Florida, United States

## Abstract

**Purpose:**

Understanding societal views toward aging is crucial for fostering inclusive communities. This study examines how participation in two community programs in Florida —a neighborhood lunch program (NLP) and a university-based lifelong learning program (LLP)—influences older adults’ perceptions of aging.

**Methods:**

A total of 193 older adults from the same county participated, with 57% from LLP. Significant demographic differences were observed: LLP participants were younger (70 vs. 77 years, p<.001), predominantly White (95% vs. 24%, p<.001), and more likely to have a college degree (92% vs. 31%, p<.001). Both groups had long-term community residency (NLP: 21.86 years; LLP: 18.96 years; non-significant).

**Results:**

Compared to LLP participants, NLP participants reported more positive perceptions of financial security, healthcare and caregiving support, family integration, and societal respect as they age. However, NLP participants perceived older adults as having less political influence. No significant differences were found between the groups regarding social participation opportunities or age-based discrimination.

**Discussion:**

These findings provide new insights into the characteristics and social perceptions of older adults in different community programs. While LLP participants viewed older adults as having greater political influence, NLP participants expressed stronger perceptions of financial and social support. The longer engagement and support structure of NLP may contribute to these more favorable views. Despite their higher socioeconomic status, LLP participants reported lower perceived societal support for older adults. Future research should explore factors influencing these perceptions, examine the benefits of NLP, and address the challenges faced by LLP participants.

